# Identification and Validation of the Pyroptosis-Related Hub Gene Signature and the Associated Regulation Axis in Diabetic Keratopathy

**DOI:** 10.1155/2024/2920694

**Published:** 2024-03-18

**Authors:** Yi Cui, Li Wang, Wentao Liang, Li Huang, Shuting Zhuang, Hong Shi, Nuo Xu, Jianzhang Hu

**Affiliations:** ^1^Department of Ophthalmology, Fujian Medical University Union Hospital, Fuzhou, Fujian, China; ^2^Department of Biochemistry, Wake Forest University School of Medicine, Winston-Salem, North Carolina 27101, USA; ^3^College of Integrated Traditional Chinese and Western Medicine, Fujian University of Traditional Chinese Medicine, Fuzhou, Fujian, China; ^4^Department of Ophthalmology, Fujian Provincial Hospital, Shengli Clinical Medical College of Fujian Medical University, Fuzhou, Fujian, China

## Abstract

**Background:**

Diabetic keratopathy (DK) poses a significant challenge in diabetes mellitus, yet its molecular pathways and effective treatments remain elusive. The aim of our research was to explore the pyroptosis-related genes in the corneal epithelium of the streptozocin-induced diabetic rats.

**Methods:**

After sixteen weeks of streptozocin intraperitoneal injection, corneal epithelium from three diabetic rats and three normal groups underwent whole-transcriptome sequencing. An integrated bioinformatics pipeline, including differentially expressed gene (DEG) identification, enrichment analysis, protein-protein interaction (PPI) network, coexpression, drug prediction, and immune deconvolution analyses, identified hub genes and key drivers in DK pathogenesis. These hub genes were subsequently validated in vivo through RT-qPCR.

**Results:**

A total of 459 DEGs were screened out from the diabetic group and nondiabetic controls. Gene Set Enrichment Analysis highlighted significant enrichment of the NOD-like receptor, Toll-like receptor, and NF-kappa B signaling pathways. Intersection of DEGs and pyroptosis-related datasets showed 33 differentially expressed pyroptosis-related genes (DEPRGs) associated with pathways such as IL-17, NOD-like receptor, TNF, and Toll-like receptor signaling. A competing endogenous RNA network comprising 16 DEPRGs, 22 lncRNAs, 13 miRNAs, and 3 circRNAs was constructed. After PPI network, five hub genes (*Nfkb1*, *Casp8*, *Traf6*, *Ptgs2*, and *Il18*) were identified as upregulated in the diabetic group, and their expression was validated by RT-qPCR in streptozocin-induced rats. Immune infiltration characterization showed that diabetic corneas owned a higher proportion of resting mast cells, activated NK cells, and memory-resting CD4 T cells. Finally, several small compounds including all-trans-retinoic acid, Chaihu Shugan San, dexamethasone, and resveratrol were suggested as potential therapies targeting these hub genes for DK.

**Conclusions:**

The identified and validated hub genes, *Nfkb1*, *Casp8*, *Traf6*, *Ptgs2*, and *Il18*, may play crucial roles in DK pathogenesis and serve as therapeutic targets.

## 1. Introduction

Diabetic keratopathy (DK) is a prevalent diabetic ocular complication, including superficial keratopathy, delayed epithelial wound healing, persistent epithelial defects, and recurrent ulceration, potentially leading to sight-threatening consequences [1, 2]. Current estimates suggest that DK occurs in 47-64% of all diabetic people [3]. With the global increase in the prevalence of diabetes, it could impose a heavy burden on public health worldwide. The pathogenesis of DK involves multiple cell types and events, such as decreased tear secretion, damaged innervation, weakened cell junctions, and impaired wound healing responses [4, 5]. Currently, the primary therapeutic strategies for DK focus on the promotion of epithelial repair with growth factors and artificial tears. The lack of explicit interventions targeting pathogenic mechanisms underscores our dedication to comprehending the intricate mechanisms associated with DK.

Hyperglycemia-induced excessive or chronic inflammation constitutes a pivotal factor in the pathogenesis of DK, leading to subsequent damage to the corneal “epineuroimmune” functional unit. As emphasized in literature [6], cell death and inflammation are intricately linked responses. Inflammasome-mediated pyroptosis is an important cell death process closely linked to diabetes and its complications, such as diabetic nephropathy [7], diabetic cardiopathy [8], and diabetic retinopathy [9]. Abnormal signals can stimulate cells to aggregate pattern recognition receptors, apoptosis speck-like protein, and pro-caspase-1 protein into a complex inflammasome. Moreover, the cleaved gasdermin D forms membrane pores, leading to cell swelling and lysis. This process further regulates the inflammatory cascade reaction by releasing proinflammatory cytokines like IL-1*β* and IL-18 [10]. In recent years, pyroptosis, a proinflammatory programmed cell death process, has emerged as a potential mechanism contributing to DK [10]. Among them, NLRP3 is the most extensively studied inflammasome within the pattern recognition receptor family. Existing literature has reported the association between the NLRP3 inflammasome and DK [11]. Local application of recombinant IL-receptor antagonist has been shown to alleviate impaired diabetic corneal wound healing [12], while subconjunctival administration of MCC950, a selective inhibitor of the NLRP3 inflammasome, has been found to expedite diabetic corneal wound closure and nerve regeneration [13]. Based on the finding above, we hypothesize that more pyroptosis-related key genes may participate in the pathogenesis of DK. However, no research has analyzed the pyroptosis-related gene profile. Therefore, establishing a system focused on pyroptosis-related genes is crucial for understanding the pathogenic mechanisms and treatment targets of DK.

In order to better understand the mechanisms of pyroptosis in DK, we employed data mining and analysis techniques to screen differentially expressed genes in the streptozocin- (STZ-) induced diabetic rat model in order to gain a deeper understanding of the underlying mechanisms of DK. Through the investigation and validation of hub differentially expressed pyroptosis-related genes, we were able to identify potential key players in the pathogenesis of DK. Our findings not only supplement existing research but also provide a reference for pyroptosis as a therapeutic target for DK.

## 2. Materials and Methods

### 2.1. Animal Study

Six male Brown Norway rats weighing 150-200 g from Slaccas, China, were used in this study. The experiments were conducted in strict accordance with the Animal Research: Reporting of In Vivo Experiments (ARRIVE) guidelines for the Use of Animals in Ophthalmic and Vision Research. The animal study was approved by the Fujian Medical University Animal Ethics Committee prior to initiation, and every effort was made to minimize any discomfort or harm to the animals involved. Animals were kept in controlled environmental conditions, including a 12-hour light and dark cycle, constant temperature and humidity, and ad libitum access to food and water. Diabetes induction was performed as previously described [4, 14]. In brief, rats were randomly divided into two groups. Streptozocin (STZ, Sigma-Aldrich, USA) was administered by a single intraperitoneal injection at 50 mg/kg to induce diabetes in rats, while citrate buffer was injected for the control group. Glucose blood readings (Ascensia Contour glucometer, Bayer Diabetes Care, USA) above 16.67 mmol/l were considered diabetic models in this study.

A Cochet-Bonnet esthesiometer (Luneau Ophtalmologie, France) was used to assess corneal sensitivity [15]. Briefly, rats were presented with a monofilament at varying lengths (6.0-0.5 cm) while unanesthetized to elicit a blink response. The longest filament length resulting in a positive response was considered the threshold of corneal sensitivity, which was verified twice. A low score indicating the absence or loss of corneal sensitivity was considered a model of DK, which was conducted in this study.

### 2.2. Sequence Analysis and Identification of Pyroptosis-Related Genes

After inducing diabetes for 16 weeks, the full-thickness cornea was meticulously trimmed off from the eyeball using surgical scissors under a microscope. Subsequently, the cornea was oriented with the epithelial side up, and the corneal epithelium was delicately scraped using a razor blade.

Total RNA was extracted from the corneal epithelium of six corneas from STZ-induced diabetic rats and six corneas from normal controls and subjected to whole-transcriptome sequencing analysis. The quantitative results can be obtained from the GEO DataSets database (accession number: GSE227165). A list of 1539 pyroptosis-related genes was curated from the MSigDB database [16] and the GeneCards database [17].

### 2.3. Data Preprocessing and Assessment of Differentially Expressed Genes (DEGs)

The data obtained from the whole-transcriptome sequencing analysis were log-transformed (base = 2) and normalized by the “affy” R package. Principal component analysis was applied to these data, and the results were plotted using the “ggplot2” package. The DEGs between diabetic corneal epithelial samples and normal samples were identified using the “limma” package [18]. The differentially expressed mRNAs (DEmRNAs), differentially expressed microRNAs (DEmiRNAs), differentially expressed long noncoding RNAs (DElncRNAs), and differentially expressed circular RNAs (DEcircRNAs) were screened uniformly following the criteria of |log2FC| > log2(1.2) and adjusted *p* value < 0.05. Volcano plots and heatmaps visualized the aforementioned DEGs with the “ggplot2” and “ComplexHeatmap” package [19], respectively.

To assess the effect of pyroptosis-related genes (PRGs) on DK, we intersected the PRGs with DEmRNAs to identify differently expressed pyroptosis-related genes (DEPRGs) by using the “ggVennDiagram” package [20]. The location of DEPRGs on rats' 21 chromosomes was drawn using the “RCircos” package [21]. In addition, the volcano plot and circle heatmap were also produced by the “ggplot2” package.

### 2.4. Establishment of Competing Endogenous RNAs (ceRNA) Networks

To investigate the hypothesis that lncRNAs or circRNAs could indirectly regulate mRNA expression by competing with miRNA as a natural sponge, the ceRNA network [22, 23] was constructed by the following steps: (1) the sequences of circRNAs and miRNAs were downloaded from circBase [24] and miRbase [25]; then, miRanda (https://www.‍miranda.‍org) was used to predict the interaction between circRNAs and miRNAs. (2) The function of lncRNA-mRNA was achieved in cis or in trans by diverse mechanisms [26]. (3) miRTarBase [27], miRecords [28], and TarBase [29] were used to forecast the validated target genes of miRNA. (4) RNA nodes that did not interact with other RNAs were removed. The generated lncRNA-mRNA-miRNA-circRNA networks were visualized by Cytoscape software [30] (version 3.7.0).

### 2.5. Functional Enrichment Analysis

With the “clusterProfiler” and “org.Rn.eg.db” (Genome wide annotation for Rat) packages, GSEA [31] was adopted to assess the distribution of the DEGs in ranked genes to determine their contributions to phenotype. The adj. *p* value, gene ratio, and normalized enrichment score (NES) were used to sort the pathways enriched in each phenotype with the “dotplot” package. A false discovery rate (FDR) < 0.25 and an adj. *p* value < 0.05 were considered significant enrichment.

To further explore the potential functional annotation and pathway attributions of DEPRGs, Gene Ontology (GO) [32] annotations were conducted to characterize biological properties, including biological process (BP), cellular component (CC), and molecular function (MF). Pathway enrichment was also undertaken with the Kyoto Encyclopedia of Genes and Genomes (KEGG) [33]. Both GO and KEGG were performed using the “clusterProfiler” package [34] and visualized with the “GOplot” package [35]. *p* < 0.05 was considered statistically significant for screening.

### 2.6. Construction of the Protein-Protein Interaction (PPI) Network and Identification of Hub Genes

The PPI networks of DEPRGs were constructed by the Search Tool for the Retrieval of Interacting Genes (STRING [36], version 11.5) online database with a combined confidence score ≥ 0.4 set as the filter condition.

Through the clusters devised by Molecular Complex Detection (MCODE) [37] in Cytoscape, key modules of the PPI network were established in line with the threshold of degree cutoff = 2, *K*‐core = 2, and node score cutoff = 0.2. Correlations between key module expressions were calculated using the nonparametric Spearman method with the “circlize” package [38].

To further narrow down the list of candidate genes, the cytoHubba [39] plugin app was conducted to explore hub genes from preloaded key modules via 10 topological algorithms, including Maximal Clique Centrality (MCC), Density of Maximum Neighborhood Component (DMNC), Maximum Neighborhood Component (MNC), Degree, Edge Percolated Component (EPC), Bottleneck, EcCentricity, Closeness, Radiality, and Stress. The final list of hub genes was screened by taking the intersection of different algorithms.

The “GOSemSim” package [40] was used to calculate the functional correlations between the hub genes linked in DK. This approach determines the likelihood of a gene being expressed by assessing its functional correlation with other genes in a pathway. The analysis was performed to identify critical genes associated with DK.

### 2.7. Immune Infiltration Analysis

To identify the immune cells in diabetic and control samples, we applied the CIBERSORTx deconvolution algorithm, which integrates labeled cell signatures derived from diverse sources, to calculate the proportion of the LM22 signature matrix based on the principle of linear support vector regression [41, 42]. We then used the Spearman correlation to determine the correlations between different immune cell types and the correlation between hub genes and significant immune cells using the “corrplot” package.

### 2.8. Prediction of Small Compounds Targeted Analysis of Hub Genes

MiRNet [43] is a miRNA-centric network visual analytic platform that contains information about miRNA-target interactions by integrating existing knowledge with users' data. miRNet was used to predict the potential downstream DEmiRNAs of candidate hug genes and further identify DEmiRNAs targeting small compounds. The hub gene-DEmiRNA-small compound networks were established by the “ggalluvial” package.

### 2.9. Validation of Characteristic Genes

The corneal epithelial samples were obtained from STZ-induced diabetic rats and nondiabetic controls. Total RNA was extracted, and cDNA was synthesized as previously described [44]. The quantitative real-time PCR (qRT-PCR) assays were conducted in triplicate using 3 independent sets of cDNA denaturation at 95°C for 10 seconds, annealing at 58°C for 30 seconds, and elongation at 72°C for 30 seconds on an Agilent Stratagene Mx3000P QPCR System. The comparative 2-*ΔΔ*Ct method was used to calculate the relative expression values, which were normalized to *β*-actin as a control. The primer sequences used are listed in Supplementary Table 1.

### 2.10. Statistics Analysis

All data processing and analysis were performed using the R software (version 4.1.0). PCR data were analyzed using SPSS 23.0 and compared using Student's *t*-test. All statistical *p* values were two-sided, and *p* < 0.05 was considered to be statistically significant.

## 3. Results

### 3.1. Establishment of the DK Model

Following STZ injection, blood glucose levels and corneal nerve sensitivity were assessed every four weeks (supplementary Figure 1). Blood glucose levels in the diabetic rat group consistently remained above 16.67 mmol/l, a statistically significant elevation compared to the normal control group. Corneal nerve sensitivity showed a mild decline at 4 and 8 weeks postinjection. A notable and statistically significant decline in corneal sensitivity was observed at both 12 and 16 weeks post-STZ injection. Thus, diabetic rats at 16 weeks postinjection conformed to the diabetic keratopathy model. Subsequently, corneal epithelial tissue was harvested for further analysis.

### 3.2. Identification of Differentially Expressed Genes (DEGs)

**Figure 1:**
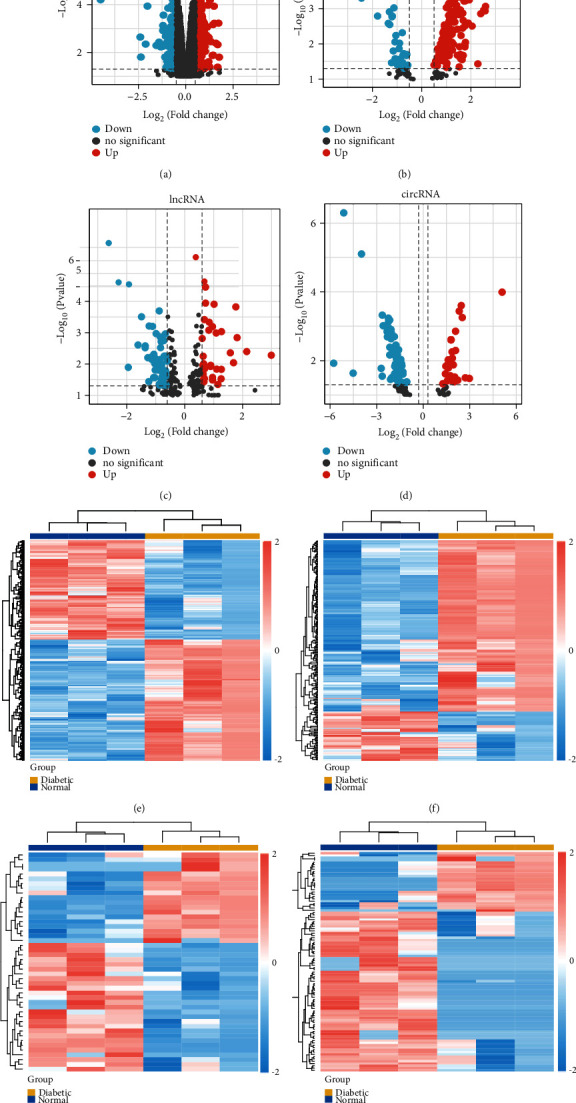
Differentially expressed genes between diabetic and normal samples. (a–d) The volcano plots indicate DEmRNAs, DEmiRNAs, DElncRNAs, and DEcircRNAs based on the fold changes > log2(1.2) and the adjusted *p* value < 0.05. Red dots indicate upregulation genes, blue dots indicate downregulation genes, and gray dots represent other genes that are not differentially expressed. (e–h) The heatmap also describes DEmRNAs, DEmiRNAs, DElncRNAs, and DEcircRNAs between different groups. The color scale represents the relative expression levels of the DEGs, where red indicates higher expression and blue indicates lower expression. DEmRNAs: differentially expressed mRNAs; DEmiRNAs: differentially expressed microRNAs; DElncRNAs: differentially expressed long noncoding RNAs; DEcircRNAs: differentially expressed circular RNAs.

The flowchart of the analysis procedure is shown in Supplementary Figure 1. The PCA exhibited a clear picture of the diabetic and normal samples (Supplementary Figure 2). Following the aforementioned threshing, a total of 459 DEmRNAs (278 upregulated and 181 downregulated mRNAs), 198 DEmiRNAs (173 upregulated and 25 downregulated miRNAs), 88 DElncRNAs (35 upregulated and 53 downregulated lncRNAs), and 96 DEcircRNAs (26 upregulated and 70 downregulated circRNAs) were sorted out from the diabetic group and normal group. The distribution of DEmRNAs, DEmiRNAs, DElncRNAs, and DEcircRNAs was illustrated by volcano plots and heatmaps, respectively (Figure 1).

### 3.3. Establishment of the Gene Set Enrichment Analysis (GSEA) of the DEmRNAs

**Figure 2:**
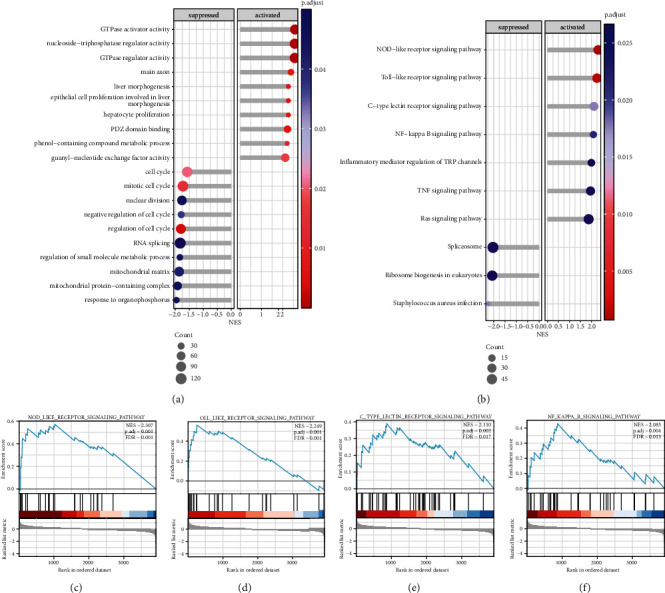
The results of Gene Set Enrichment Analysis (GSEA). (a) The top gene sets that were significantly upregulated and downregulated based on enrichment analysis in the Gene Ontology. (b) The top gene sets were significantly upregulated and downregulated based on enrichment analysis in the pathways. (c–f) GSEA plots showed the top four marked enriched pathway. Each run was performed with 1,000 permutations.

As shown in Figure 2(a), GTPase activator activity, nucleoside-triphosphatase regulator activity, and GTPase regulator activity were activated in the diabetic groups, while negative regulation of response to organophosphorus, mitochondrial protein-containing complex, and mitochondrial matrix were suppressed. In addition, significant enrichment was observed in several signaling pathways, including the NOD-like receptor signaling pathway, Toll-like receptor signaling pathway, C-type lectin receptor signaling pathway, NF-kappa B signaling pathway, and inflammatory mediator regulation of TRP channels, in the diabetic group compared to the normal group (*p* < 0.05, Figure 2(b)). The important enriched pathway of the detailed GSEA is presented in Figures 2(c)–2(f). Complete result lists from the GSEA are provided in Supplementary Table 2.

### 3.4. Identification of Differentially Expressed Pyroptosis-Related Genes (DEPRGs)

**Figure 3:**
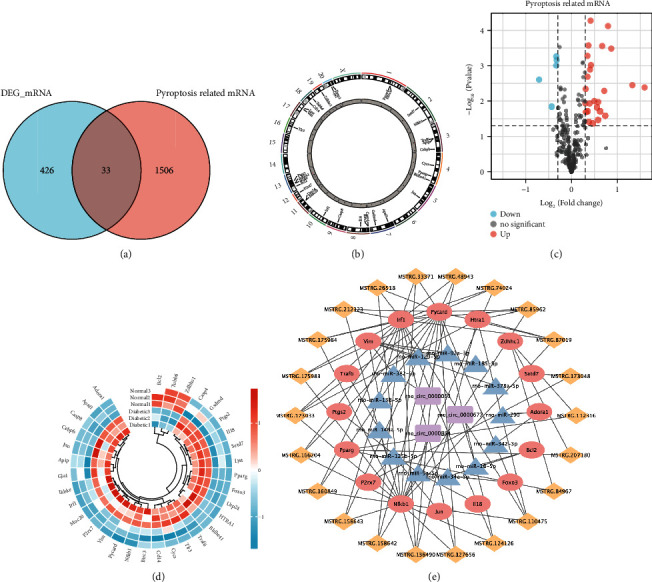
Identification of the differentially expressed pyroptosis-related genes (DEPRGs). (a) Venn diagram shows the overlap between the pyroptosis-related genes (in red) and DEmRNAs (in blue). (b) Circos plot depicts the chromosome positions of the 33 DEPRGs on the 21 chromosomes of rats. (c, d) The volcano and circle heatmap illustrate the upregulated and downregulated DEPRGs in the diabetic and control groups. Red represents higher expression, while blue represents lower expression. (e) Construction of the lncRNA-mRNA-miRNA-circRNA regulatory network. The colors pink, blue, yellow, and purple denote DEPRGs, DEmiRNAs, DElncRNAs, and DEcircRNAs, respectively. DEmiRNAs: differentially expressed microRNAs; DElncRNAs: differentially expressed long noncoding RNAs; DEcircRNAs: differentially expressed circular RNAs.

To evaluate the expression patterns of pyroptosis-related genes (PRGs), we overlapped the DEmRNAs with PRGs to obtain 33 differentially expressed pyroptosis-related genes (DEPRGs) for further analyses (Figure 3(a) and Supplementary Table 3). Most of these genes were annotated on chromosomes 3, 4, 8, and 13 (Figure 3(b)). Of the DEPRGs identified, 30 were upregulated and 3 were downregulated, as shown in the volcano plot and circle heatmap (Figures 3(c) and 3(d)).

### 3.5. Competing Endogenous (ceRNA) Networks Based on DEPRGs

Based on the ceRNA hypothesis, we constructed a lncRNA-mRNA-miRNA-circRNA network involving DEPRGs and took the intersection with our DEPRG list described above. A total of 17 mRNA-miRNA pairs (10 DEPRGs and 10 DEmiRNAs), 60 mRNA-lncRNA pairs (14 DEPRGs and 22 DElncRNAs), and 3 miRNA-circRNA pairs (3 DEmiRNAs and 3 DEcircRNAs) were identified to predict the interaction. This network was visualized by representing the distribution of interactions through Cytoscape (Figure 3(e)).

### 3.6. Functional Enrichment Analysis Based on DEPRGs

**Figure 4:**
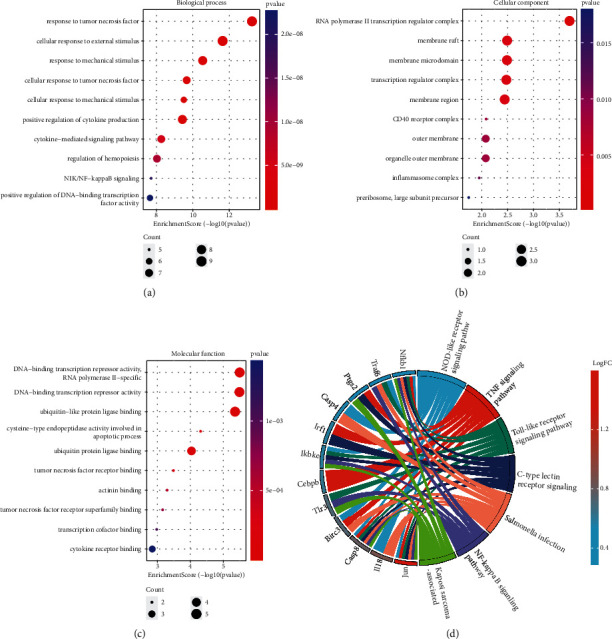
Functional enrichment analysis of the differentially expressed pyroptosis-related genes (DEPRGs). Bubble plot shows Gene Ontology annotation analysis of DEPRGs, including (a) biological process (BP), (b) cellular component (CC), and (c) molecular function (MF). (d) Chord plot illustrates the results of Kyoto Encyclopedia of Genes and Genomes (KEGG) pathway analysis of DEPRGs.

To elucidate the biological functions and pathways of DEPRGs, GO and KEGG analyses were performed. In the biological process category, the results revealed that the DEPRGs mainly affect response to tumor necrosis factor, cellular response to external stimulus, cytokine-mediated signaling pathway, and NIK/NF-kappa B signaling (Figure 4(a)). In the cellular component category, the majority of DEPRGs were enriched in the RNA polymerase II transcription regulator complex, membrane raft, transcription regulator complex, and inflammasome complex (Figure 4(b)). For molecular function, the most significant entries were DNA-binding transcription repressor activity (RNA polymerase II-specific), ubiquitin-like protein ligase binding, cysteine-type endopeptidase activity involved in the apoptotic process, and tumor necrosis factor receptor binding (Figure 4(c)). Furthermore, the KEGG analysis exhibited that the most involved pathways were IL-17 signaling, NOD-like receptor signaling pathway, TNF signaling pathway, Toll-like receptor signaling pathway, and C-type lectin receptor signaling pathway (Figure 4(d)). Additional information regarding the GO and KEGG analyses is shown in Supplementary Table 4.

### 3.7. Protein-Protein Interaction (PPI) Network Construction and Hub Gene Analyses

**Figure 5:**
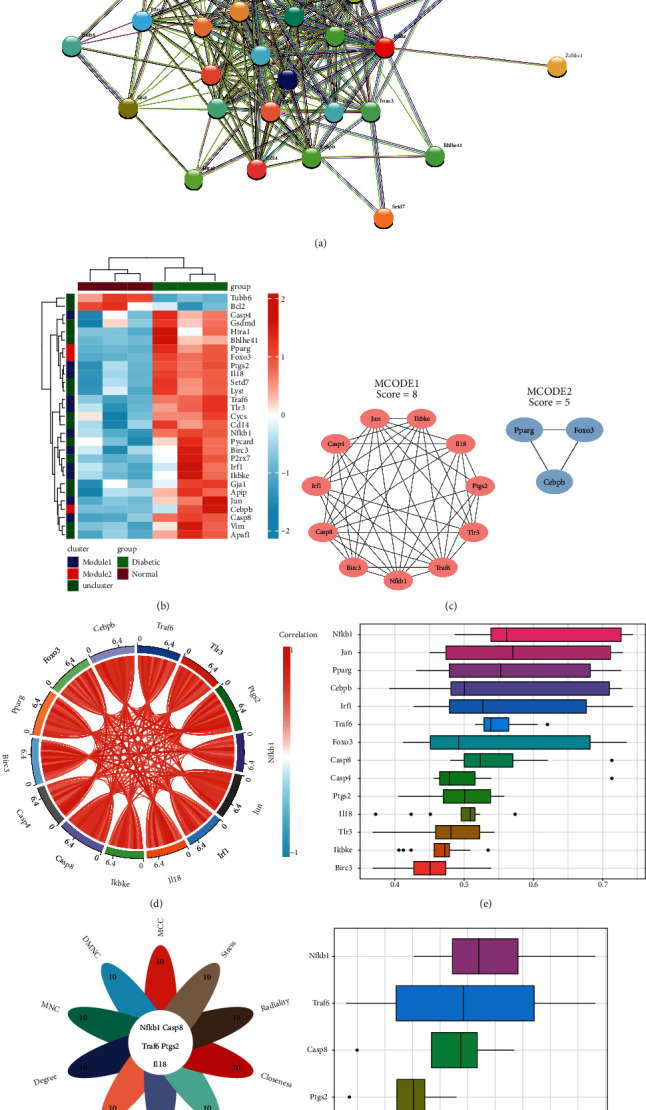
Protein-protein interaction (PPI) network integration and identification of hub genes. (a) Visualization of DEPRGs obtained from STRING database with interaction scores > 0.4. (b) The distribution of DEPRGs that are predicted by Molecular Complex Detection. The red and blue colors represent higher and lower expression, respectively. (c) Identification of key module 1 and module 2 in the PPI network by Molecular Complex Detection. (d) The correlation analysis between the different hub genes. The red and blue colors represent positive and negative correlations, respectively. (e) The functional similarity of the hub genes using the friends analysis. *Nfkb1* showed the strongest correlation with other genes. (f) Hub genes screened by 10 algorithms from cytoHubba. (g) The functional similarity of the five hub genes using the friends analysis. DEPRGs: differentially expressed pyroptosis-related genes; MCC: Maximal Clique Centrality; DMNC: Density of Maximum Neighborhood Component; MNC: Maximum Neighborhood Component; EPC: Edge Percolated Component.

After eliminating the isolated genes without interaction, the PPI network was constructed, which contained 33 nodes and 185 edges (Figure 5(a)). The functional enrichment analysis via the STRING database indicated that these DEPRGs were significantly associated with pyroptosis, I-kappa B phosphorylation, cysteine-type endopeptidase activity involved in apoptotic process, NLRP3 inflammasome complex, and AIM2 inflammasome complex. Furthermore, the distribution of 33 DEPRGs in the different clusters was displayed in a heatmap (Figure 5(b)).

Through the MCODE plugin in Cytoscape, two key modules from the PPI network were established (Figure 5(c)). There were 11 nodes in module 1, namely, *Ptgs2*, *Il18*, *Tlr3*, *Traf6*, *Nfkb1*, *Birc3*, *Casp8*, *Irf1*, *Casp4*, *Jun*, and *Ikbke*, with the highest MCODE scores. Three genes were obtained in module 2, including *Pparg*, *Foxo3*, and *Cebpb*. The Spearman correlation analysis was performed for each gene individually (Figure 5(d)). All genes belonged to the upregulated DEPRGs, and most of them displayed a strong positive correlation. Specifically, *Traf6* and *Tlr3*, *Il18* and *Casp4*, and *Jun* and *Cebpb* showed a significantly positive correlation (*r* > 0.95, *p* < 0.01). *Nfkb1* was reflected by a positive correlation with *Irf1* and *Ikbke* (*r* > 0.99, *p* < 0.01). Moreover, we calculated the semantic similarity and found that *Nfkb1* exhibited the highest similarity among the other 13 hub genes (Figure 5(e)).

Furthermore, according to the scores with cytoHubba in Cytoscape, all key modules were ranked in the top 10 of each algorithm. The intersection of these 10 algorithms was then identified as hub genes, which included *Nfkb1*, *Casp8*, *Traf6*, *Ptgs2*, and *Il18* (Figure 5(f)). Based on the semantic similarity, *Nfkb1* exhibited the highest similarity among the other 4 hub genes (Figure 5(g)). Therefore, we decided to use these 5 genes as hub genes for subsequent analysis.

### 3.8. Immune Infiltration Landscape Analysis

**Figure 6:**
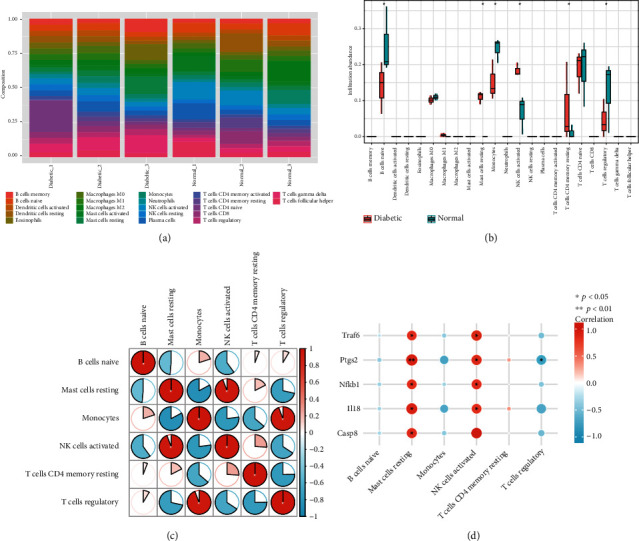
Comparison of immune cells and hub genes between diabetic and normal groups. (a) The percentage of 22 immune cell types is shown using the CIBERSORTx algorithm. (b) Box plot shows the differences in the abundance of immune cells in different groups (^∗^*p* < 0.05). (c) The correlation of significant immune cell proportions in the diabetic groups, with red indicating positive correlation and blue indicating negative correlation. (d) The correlation analysis of the significantly infiltrated immune cells and hub genes in the diabetic groups, with red indicating positive correlation and blue indicating negative correlation (^∗^*p* < 0.05, ^∗∗^*p* < 0.01).

Many pieces of evidence indicate a strong link between the immune response and DK [1, 45, 46]. Therefore, we explored the panorama of the immune microenvironment with the CIBERSORTx algorithm. The distribution of immune cells is demonstrated in Figure 6(a), revealing different fractions of immune cells. The diabetic groups showed a higher proportion of resting mast cells, activated NK cells, and memory-resting CD4 T cells than those in the normal groups. However, the levels of naïve B cells, monocytes, and regulatory T cells were significantly lower than those in the normal groups (*p* < 0.05, Figure 6(b)). The proportion of the remaining immune cells did not differ significantly between the two groups. The regulatory T cells showed significantly positive correlations with monocytes, and activated NK cells were positively correlated with resting mast cells (*p* < 0.05, Figure 6(c)). In addition, we implemented the relationship between hub gene expression and the immune cells in the diabetic groups (Figure 6(d)). The results displayed that all hub genes had strong positive correlations with resting mast cells and activated NK cells (*r* > 0.8, *p* < 0.05), while *Ptgs2* was negatively correlated with regulatory T cells (*r* = −0.82, *p* = 0.045).

### 3.9. Prediction of Hub Gene-miRNA-Small Compound Networks

**Figure 7:**
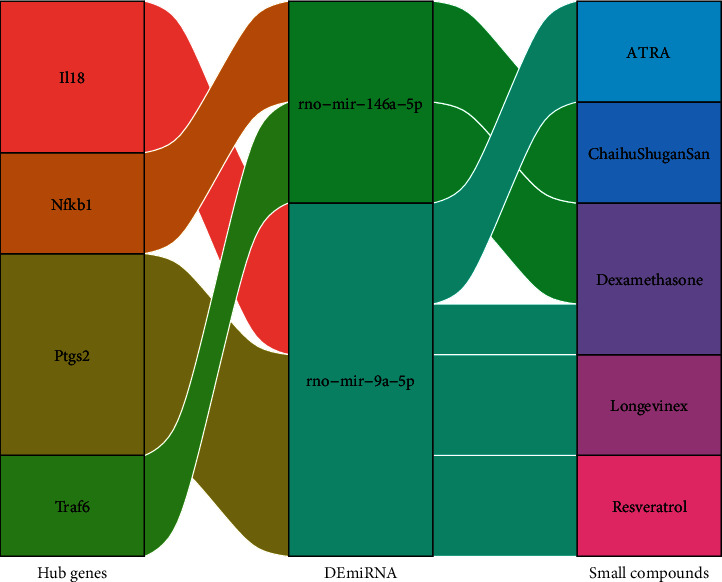
The interaction network of hub gene-DEmiRNA-small compounds. The first column represents the hub genes, the second column corresponds to the DEmiRNAs, and the third column to the small compounds. DEmiRNAs: differentially expressed microRNAs.

Target prediction revealed that numerous hub genes were potentially regulated by DEmiRNAs, and small compounds were potentially regulated by these miRNAs. We searched for a hub gene-DEmiRNA-small compound regulatory network using the miRNet 2.0 database. A total of 20 significant interactions (*p* < 0.05) were identified, which included 4 hub genes (*Il18*, *Nfkb1*, *Ptgs2*, and *Traf6*), 2 DEmiRNAs (rno-mir-146a-3p and rno-mir-9a-5p), and 5 small compounds (ATRA (all-trans-retinoic acid), Chaihu Shugan San, dexamethasone, Longevinex (modified resveratrol), and resveratrol) (Figure 7).

### 3.10. Validation of the Hub Genes

**Figure 8:**
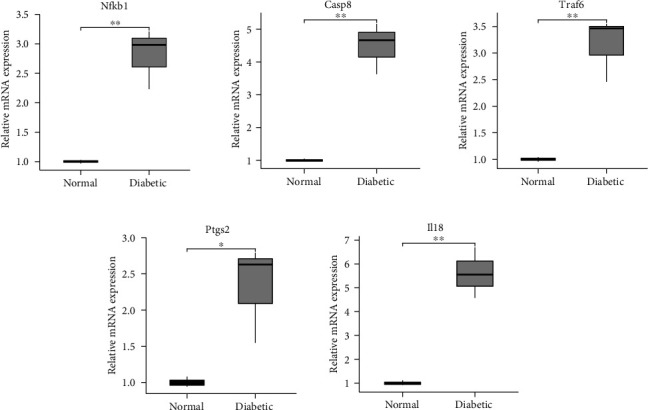
Verification of the hub genes by quantitative real-time PCR. The expression of *Nfkb1*, *Casp8*, *Traf6*, *Ptgs2*, and *Il18* was found to be significantly upregulated in diabetic corneal samples (^∗^*p* < 0.05, ^∗∗^*p* < 0.01).

The expression of five genes related to pyroptosis was analyzed by qRT-PCR in twelve corneal epithelial tissues from rats, including six normal samples and six diabetic samples. All 5 genes (*Nfkb1*, *Casp8*, *Traf6*, *Ptgs2*, and *Il18*) were significantly upregulated in diabetic corneal samples compared to normal controls (*p* < 0.05) (Figure 8), indicating that the results were reproducible and reliable.

## 4. Discussion

Diabetes mellitus is a major public health issue because of its high morbidity and mortality rate [47]. Diabetic patients are at increased risk of developing corneal complications such as recurrent erosions, delayed epithelial healing, increased endothelial cell loss, and a higher possibility of infections following cataract and refractive surgeries. It is well known that severe or chronic inflammation leads to outcomes in terms of corneal clarity, thickness, and healing [2]. To delineate the molecular alterations and explore potential disease markers associated with DK, we analyzed and identified the pyroptosis-related genes in the cornea of diabetic rats.

In this study, we found that pyroptosis-specific markers, including IL-18 and gasdermin D (GSDMD), were upregulated in the DK group. The major canonical inflammasome NLRP3 can recruit and activate the adaptor protein apoptosis-associated speck-like protein-containing CARD and caspase-1, which further cleaves GSDMD and induces the release of proinflammatory cytokines such as IL-1*β* and IL-18 [48]. GSDMD is a physiological substrate of the canonical inflammasome pathway and plays a central role in the impairment of diabetic wound healing [49]. IL-18, produced by human corneal epithelial and stromal fibroblast cells [49–51], could mediate a series of intracellular signal transduction including the activation of NF-*κ*B, which was also elevated in the DK group, and promote the inflammatory response. Recent research found that increased IL-18 was involved in the pathogenesis of diabetic cardiomyopathy [52] and expressed in the renal and retinas of the STZ-induced diabetic rats [53, 54]. Moreover, the observation of the cell viability and ultrastructure of retinal stem cells revealed that IL-18 induces pyroptosis protein expression in retinal cells [55]. These findings extend the impact of IL-18 beyond DK, suggesting potential research avenues. We also found that FOXO3 was upregulated in the DK group. This gene belongs to the forkhead family of transcription factors, which are characterized by a distinct forkhead domain. It encodes a protein that functions as a trigger for apoptosis through the expression of genes necessary for cell death [56, 57]. Recently, activating FOXO3 was shown to promote diabetic corneal epithelial wound healing [58]. It unravels FOXO3's role in DK pathogenesis, and its interactions may reveal novel therapeutic or diagnostic prospects.

In diabetic corneas, epithelium and immune cells are altered during wound healing. Hence, exploring the immune microenvironment is critical for understanding the pathophysiology of DK. In this study, we found that the diabetic groups owned a higher proportion of NK cells, CD4+ T cells, and mast cells. Previous evidence supported the idea that NK cells could modulate the inflammatory response to corneal epithelial abrasion and promote wound healing [59]. When activated, NK cells induce apoptosis of the target cells by secreting proinflammatory cytokines such as interferon-gamma, tumor necrosis factor, granulocyte-macrophage colony-stimulating factor, and macrophage inflammatory proteins 1a and 1b. Mast cells are bone marrow progenitor-derived immune cells that are increased in diabetic patients and animal models of diabetes, and they have been shown to modulate local inflammation and improve diabetic wound healing [60, 61]. Tregs play a critical role in maintaining self-tolerance and preventing the onset of autoimmune diseases [62]. Loss of Treg function can result in chronic inflammation [63], whereas excess activation of Tregs increases the risk of ocular pathologies ranging from delayed epithelial wound healing and chronic pain to recurrent erosions [64]. These conditions can further lead to corneal scarring and thinning.

To further explore the enriched pathways involved in the development of DK, we found several enriched pathways in the diabetic group, including the IL-17 signaling pathway, NOD-like receptor signaling pathway, TNF signaling pathway, and Toll-like receptor signaling pathway. Previous studies have demonstrated that the expression of NLRP3, NF-*κ*B p65, and p-NF-*κ*B protein and mRNA was significantly enhanced in the diabetic group [65]. Also, high concentrations of TNF-*α* and the activated IL-17 signaling pathway have been shown to play an essential role in STZ-induced diabetic corneal epithelium and diabetic nephropathy [66, 67]. The diabetic corneas had significantly increased Toll-like receptor 4 expression, which was involved in corneal wound and nerve healing [68]. These findings collectively suggested that inflammation and immune response are critical factors involved in the pyroptosis-mediated pathogenesis of DK.

Through building hub gene-miRNA-small compound networks, we uncovered some potential small compounds like resveratrol and dexamethasone that may have therapeutic benefits in DK. Resveratrol, an antioxidant phytogenic substance, has been reported to control NF-*κ*B activation and modulate inflammatory gene expression through the Toll-like receptor pathway in epithelial cells [69, 70]. Similarly, dexamethasone, a glucocorticoid receptor (GR) agonist, may reversed neuroinflammation through the GR/NF-*κ*B signaling pathway in diabetic rats [71]. However, topical steroid use is usually not recommended for corneal epithelial defects due to the potential to delay healing [72]. Further experiments are warranted to investigate the efficacy of these potential candidates in the treatment of DK.

Although our current study improved the understanding of the relationship between hub genes and DK, there were still some limitations. First, the sample size used in the analysis was relatively small, and this may have impacted the results obtained. Additionally, although we were able to validate the expression of hub genes, we did not comprehensively evaluate additional important signaling pathways or direct mechanisms of hub genes and potential compounds involved in the process of DK. In the future, our focus will be exploring the protective effects of diverse compounds on DK to pave the way for innovative treatment strategies. Furthermore, the current study was performed using RNA sequencing from corneal tissue of diabetic rats, and therefore, we were unable to illustrate the expression of hub genes at the cellular level. Further studies could consider using single-cell sequencing to explore the direct mechanisms underlying the relationship between hub genes and DK.

## 5. Conclusions

In conclusion, we conducted a thorough and systematic bioinformatics analysis, identifying a hub gene signature related to pyroptosis that includes five genes (Nfkb1, Casp8, Traf6, Ptgs2, and Il18) and investigating potential compound treatment approaches for DK. Additionally, our research specifically targets transcriptomic analysis of the corneal epithelium under STZ-induced diabetic conditions, filling a notable gap in previous investigations in this area. The findings offer promising insights into DK's underlying mechanisms and propose a novel strategy for diagnosis and treatment. Further studies are essential to validate these findings.

## Data Availability

The datasets generated and/or analyzed during the current study are available from the corresponding author upon reasonable request. And the datasets presented in this study can be found in online repositories. The names of the repository/repositories and accession number(s) can be found in https://www.ncbi.nlm.nih.gov/geo/query/acc.cgi?acc=GSE227165.

## Supplementary Materials

Supplementary Materials Supplementary Figure 1: changes in blood glucose level and corneal sensitivity at four-week intervals after streptozocin injection. Supplementary Figure 2: flowchart to illustrate the analysis process of the present study. Supplementary Figure 3: principal component analysis on different groups. Principal component analysis (PCA) of complete genome data from corneal epithelial samples depicts the perfectly discriminated between the diabetic and control samples. Supplementary Table 1: primer sequences. Supplementary Table 2: Gene Set Enrichment Analysis (GSEA) enrichments. Supplementary Table 3: the list of differentially expressed pyroptosis-related genes (DEPRGs). Supplementary Table 4: Gene Ontology (GO) terms and Kyoto Encyclopedia of Genes and Genomes (KEGG) pathway enrichments.
