# Supplementary material for: Identification and Validation of the Pyroptosis-Related Hub Gene Signature and the Associated Regulation Axis in Diabetic Keratopathy
Source: J Diabetes Res. 2024 Mar 18;2024:2920694. doi: 10.1155/2024/2920694 (PMC10963115; doi:10.1155/2024/2920694)
Supplement: Supplementary Materials — Supplementary Figure 1: changes in blood glucose level and corneal sensitivity at four-week intervals after streptozocin injection. Supplementary Figure 2: flowchart to illustrate the analysis process of the present study. Supplementary Figure 3: principal component analysis on different groups. Principal component analysis (PCA) of complete genome data from corneal epithelial samples depicts the perfectly discriminated between the diabetic and control samples. Supplementary Table 1: primer sequences. Supplementary Table 2: Gene Set Enrichment Analysis (GSEA) enrichments. Supplementary Table 3: the list of differentially expressed pyroptosis-related genes (DEPRGs). Supplementary Table 4: Gene Ontology (GO) terms and Kyoto Encyclopedia of Genes and Genomes (KEGG) pathway enrichments. [file 2920694.f1.docx]

**Supplementary Figure 1**


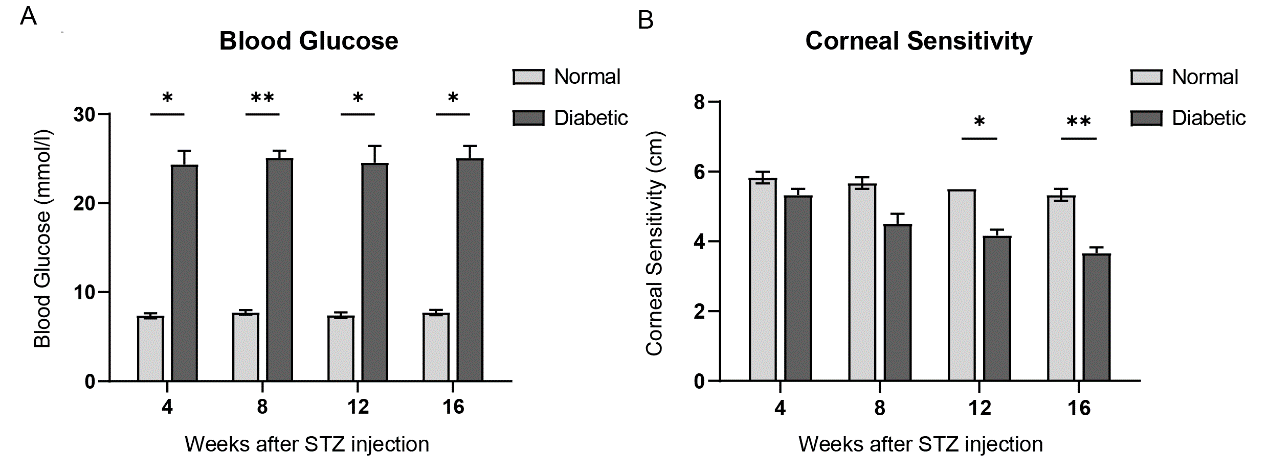


**Supplementary Figure 1** Changes in blood glucose level and corneal sensitivity at four-week intervals after streptozocin injection. (A) Blood glucose levels in the diabetic rats were significantly higher than that in normal rats, and blood glucose of the diabetic groups maintained greater than 16.67mmol/l through the experimental period (n = 3 per group). (B) Corneal sensitivity exhibited a gradual decline in the diabetic rats, with statistical differences becoming significant after 12 weeks of streptozocin injection (n = 3 per group). *P < 0.05, **P < 0.01.

**Supplementary Figure 2**

**
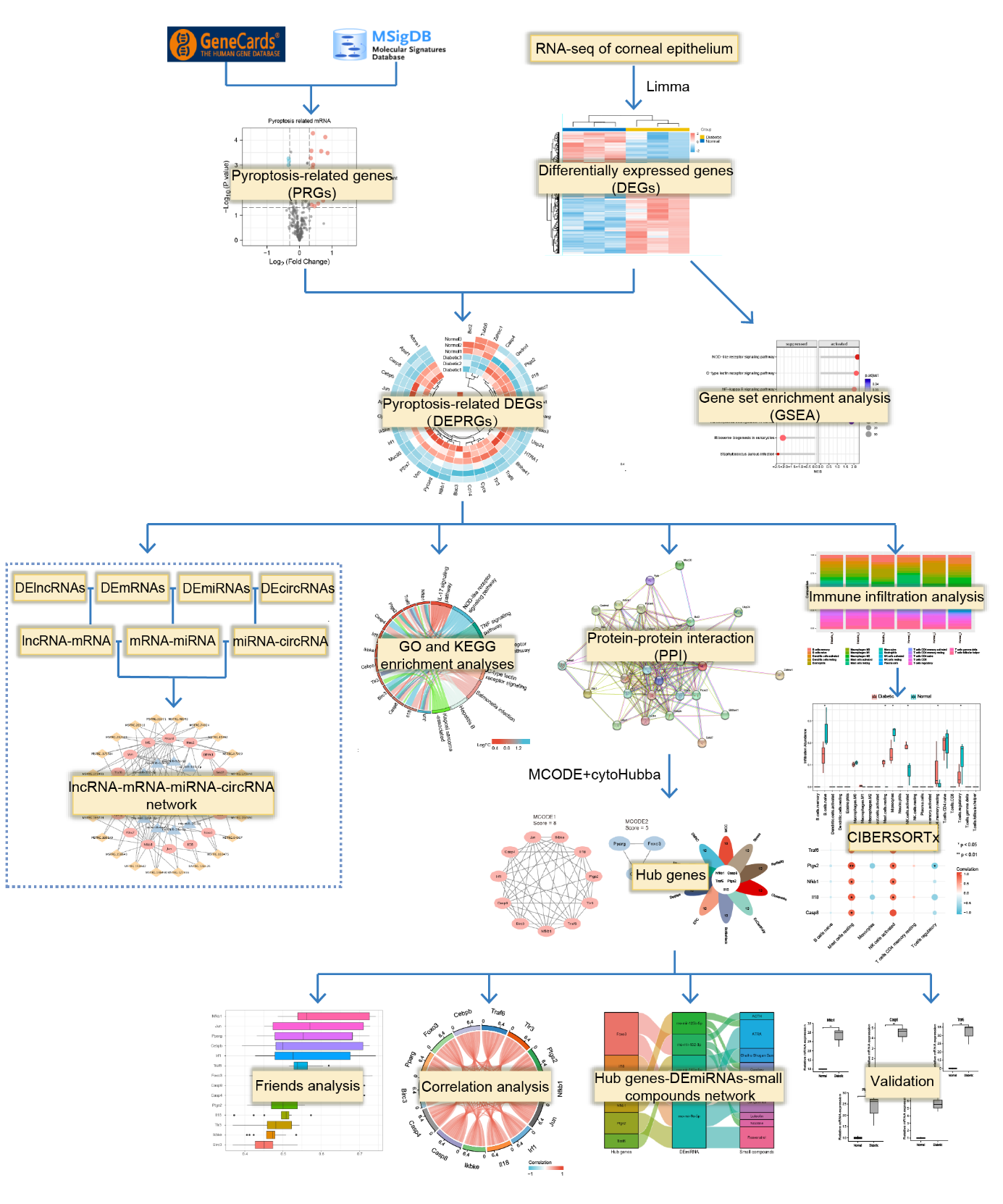
**

**Supplementary Figure 2** Flow chart to illustrate the analysis process of the present study.

**Supplementary Figure 3**

**
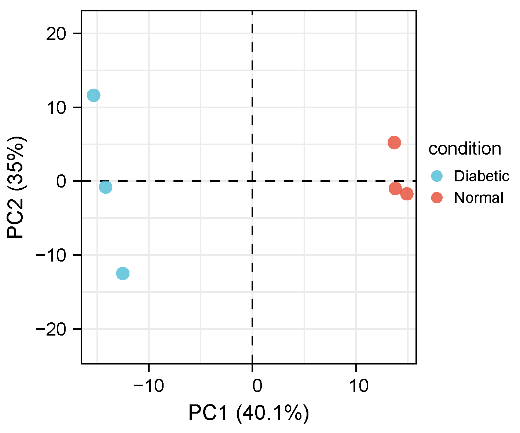
**

**Supplementary Figure 3** Principal component analysis on different groups. Principal component analysis (PCA) of complete genome data from corneal epithelial samples depicts the perfectly discriminated between the diabetic and control samples.

**Supplementary Table 1 Primer sequences**

| **Gene Symbol** | **Primer direction** | **Sequences (5’to 3’)** |
| --- | --- | --- |
| *Traf6* | Forward | ACATCCCTGAGCTGTCAAGC |
|  | Reverse | AGAGTTCAAAGGGGGCACTG |
| *Ptgs2* | Forward | ACCCCCTGCTGCCCGACACCT |
|  | Reverse | CCAGCAACCCGGCCAGCAATC |
| *Nfkb1* | Forward | TCCACTGTCTGCCTCTCTCGTC |
|  | Reverse | GCCTTCAATAGGTCCTTCCTGC |
| *Il18* | Forward | GACTCTTGCGTCAACTTCAAGG |
|  | Reverse | CAGGCTGTCTTTTGTCAACGA |
| *Casp8* | Forward | AACGTCTGGGCAACGAAGAAC |
|  | Reverse | ACGAGCTCCTTCAGGAAGGAC |
| *β-actin* | Forward | AGGGAAATCGTGCGTGAC |
|  | Reverse | CATACCCAAGAAGGAAGGCT |

**Supplementary Table 2 Gene set enrichment analysis (GSEA) enrichments**

| Description | NES | P-value |
| --- | --- | --- |
| GTPase activator activity | 2.582 | 5.974E-10 |
| nucleoside-triphosphatase regulator activity | 2.567 | 4.764E-10 |
| GTPase regulator activity | 2.567 | 4.764E-10 |
| main axon | 2.386 | 5.995E-05 |
| liver morphogenesis | 2.266 | 5.470E-05 |
| epithelial cell proliferation involved in liver morphogenesis | 2.266 | 5.470E-05 |
| hepatocyte proliferation | 2.266 | 5.470E-05 |
| PDZ domain binding | 2.224 | 6.478E-05 |
| phenol-containing compound metabolic process | 2.209 | 1.281E-04 |
| guanyl-nucleotide exchange factor activity | 2.118 | 2.459E-04 |
| cell cycle | -1.570 | 3.390E-04 |
| mitotic cell cycle | -1.724 | 1.910E-04 |
| nuclear division | -1.760 | 1.610E-03 |
| negative regulation of cell cycle | -1.787 | 9.782E-04 |
| regulation of cell cycle | -1.799 | 1.475E-05 |
| RNA splicing | -1.825 | 1.671E-03 |
| regulation of small molecule metabolic process | -1.837 | 1.477E-03 |
| mitochondrial matrix | -1.860 | 1.171E-03 |
| mitochondrial protein-containing complex | -1.914 | 1.436E-03 |
| response to organophosphorus | -1.957 | 1.450E-03 |
| NOD-like receptor signaling pathway | 2.307 | 3.150E-06 |
| Toll-like receptor signaling pathway | 2.249 | 1.030E-05 |
| C-type lectin receptor signaling pathway | 2.110 | 2.810E-04 |
| NF-kappa B signaling pathway | 2.085 | 4.390E-04 |
| Inflammatory mediator regulation of TRP channels | 1.994 | 9.340E-04 |
| TNF signaling pathway | 1.953 | 1.091E-03 |
| Ras signaling pathway | 1.870 | 8.500E-04 |
| Spliceosome | -2.026 | 1.098E-03 |
| Ribosome biogenesis in eukaryotes | -2.062 | 8.580E-04 |
| Staphylococcus aureus infection | -2.232 | 2.900E-04 |

NES, normalized enrichment score.

**Supplementary Table 3 The list of differentially expressed pyroptosis-related genes (DEPRGs)**

| Gene | Description | logFC | adj.P.Val |
| --- | --- | --- | --- |
| *Traf6* | tnf receptor associated factor 6 | 0.334 | 5.500E-04 |
| *Tlr3* | toll like receptor 3 | 0.709 | 2.480E-03 |
| *Ptgs2* | prostaglandin-endoperoxide synthase 2 | 0.424 | 9.600E-04 |
| *Nfkb1* | nuclear factor kappa b subunit 1 | 0.292 | 1.550E-03 |
| *Jun* | jun proto-oncogene, ap-1 transcription factor subunit | 1.595 | 4.130E-03 |
| *Irf1* | interferon regulatory factor 1 | 0.578 | 3.413E-02 |
| *Il18* | interleukin 18 | 0.867 | 3.300E-04 |
| *Ikbke* | inhibitor of nuclear factor kappa b kinase subunit epsilon | 0.582 | 1.068E-02 |
| *Casp8* | caspase 8 | 0.796 | 8.000E-05 |
| *Casp4* | caspase 4 | 0.556 | 1.521E-02 |
| *Birc3* | baculoviral iap repeat containing 3 | 0.730 | 2.580E-02 |
| *Pparg* | peroxisome proliferator activated receptor gamma | 0.669 | 2.800E-04 |
| *Foxo3* | forkhead box o3 | 0.415 | 5.000E-05 |
| *Cebpb* | ccaat enhancer binding protein beta | 0.620 | 1.948E-02 |
| *Vim* | vimentin | 0.714 | 5.180E-03 |
| *Tubb6* | tubulin beta 6 class v | -0.325 | 6.900E-04 |
| *Setd7* | set domain containing 7, histone lysine methyltransferase | 0.348 | 2.050E-03 |
| *Pycard* | pyd and card domain containing | 0.351 | 1.938E-02 |
| *P2rx7* | purinergic receptor p2x 7 | 0.297 | 1.042E-02 |
| *Lyst* | lysosomal trafficking regulator | 0.310 | 4.570E-03 |
| *Htra1* | htra serine peptidase 1 | 0.395 | 1.179E-02 |
| *Gsdmd* | gasdermin d | 0.377 | 3.838E-02 |
| *Gja1* | gap junction protein alpha 1 | 0.280 | 3.531E-02 |
| *Cycs* | cytochrome c, somatic | 0.318 | 2.026E-02 |
| *Cd14* | cd14 molecule | 1.328 | 3.560E-03 |
| *Bhlhe41* | basic helix-loop-helix family member e41 | 0.496 | 1.004E-02 |
| *Bcl2* | bcl2 apoptosis regulator | -0.430 | 1.459E-02 |
| *Apip* | apaf1 interacting protein | 0.301 | 1.167E-02 |
| *Apaf1* | apoptotic peptidase activating factor 1 | 0.405 | 1.290E-03 |
| *Muc20* | mucin 20, cell surface associated | 0.465 | 4.298E-02 |
| *Usp24* | ubiquitin specific peptidase 24 | 0.369 | 2.700E-04 |
| *Adora1* | adenosine a1 receptor | 0.347 | 5.300E-04 |
| *Zdhhc1* | zinc finger dhhc-type containing 1 | -0.335 | 9.900E-04 |

**Supplementary Table 4 Gene Ontology (GO) terms and Kyoto Encyclopedia of Genes and Genomes (KEGG) pathway enrichments**

| Ontology | ID | Description | P.value |
| --- | --- | --- | --- |
| BP | GO:0034612 | response to tumor necrosis factor | 6.920E-11 |
| BP | GO:0071496 | cellular response to external stimulus | 1.421E-09 |
| BP | GO:0009612 | response to mechanical stimulus | 1.179E-08 |
| BP | GO:0071356 | cellular response to tumor necrosis factor | 6.698E-08 |
| BP | GO:0071260 | cellular response to mechanical stimulus | 7.679E-08 |
| BP | GO:0001819 | positive regulation of cytokine production | 7.679E-08 |
| BP | GO:0019221 | cytokine-mediated signaling pathway | 9.509E-07 |
| BP | GO:1903706 | regulation of hemopoiesis | 1.441E-06 |
| BP | GO:0038061 | NIK/NF-kappaB signaling | 2.673E-06 |
| BP | GO:0051091 | positive regulation of DNA-binding transcription factor activity | 2.791E-06 |
| CC | GO:0090575 | RNA polymerase II transcription regulator complex | 7.853E-03 |
| CC | GO:0045121 | membrane raft | 2.817E-02 |
| CC | GO:0098857 | membrane microdomain | 2.817E-02 |
| CC | GO:0005667 | transcription regulator complex | 2.817E-02 |
| CC | GO:0098589 | membrane region | 2.817E-02 |
| CC | GO:0035631 | CD40 receptor complex | 4.088E-02 |
| CC | GO:0019867 | outer membrane | 4.088E-02 |
| CC | GO:0031968 | organelle outer membrane | 4.088E-02 |
| CC | GO:0061702 | inflammasome complex | 4.875E-02 |
| CC | GO:0030687 | preribosome, large subunit precursor | 6.554E-02 |
| MF | GO:0001227 | DNA-binding transcription repressor activity, RNA polymerase II-specific | 1.571E-04 |
| MF | GO:0001217 | DNA-binding transcription repressor activity | 1.571E-04 |
| MF | GO:0044389 | ubiquitin-like protein ligase binding | 1.571E-04 |
| MF | GO:0097153 | cysteine-type endopeptidase activity involved in apoptotic process | 1.304E-03 |
| MF | GO:0031625 | ubiquitin protein ligase binding | 1.975E-03 |
| MF | GO:0005164 | tumor necrosis factor receptor binding | 5.829E-03 |
| MF | GO:0042805 | actinin binding | 7.730E-03 |
| MF | GO:0032813 | tumor necrosis factor receptor superfamily binding | 9.273E-03 |
| MF | GO:0001221 | transcription cofactor binding | 1.244E-02 |
| MF | GO:0005126 | cytokine receptor binding | 1.492E-02 |
| KEGG | rno04621 | NOD-like receptor signaling pathway | 5.801e-11 |
| KEGG | rno04668 | TNF signaling pathway | 1.133e-10 |
| KEGG | rno04620 | Toll-like receptor signaling pathway | 3.022e-09 |
| KEGG | rno04625 | C-type lectin receptor signaling pathway | 8.403e-09 |
| KEGG | rno05132 | Salmonella infection | 3.992e-08 |
| KEGG | rno04064 | NF-kappa B signaling pathway | 6.981e-08 |
| KEGG | rno05167 | Kaposi sarcoma-associated herpesvirus infection | 4.523e-07 |

BP, biological process; CC, cellular component; MF, molecular function.
